# The role of cat eye narrowing movements in cat–human communication

**DOI:** 10.1038/s41598-020-73426-0

**Published:** 2020-10-05

**Authors:** Tasmin Humphrey, Leanne Proops, Jemma Forman, Rebecca Spooner, Karen McComb

**Affiliations:** 1 Mammal Communication and Cognition Research Group, School of Psychology, University of Sussex, Brighton, BN1 9QH UK; 2 Department of Psychology, Centre for Comparative and Evolutionary Psychology, University of Portsmouth, Portsmouth, PO1 2DY UK

**Keywords:** Psychology, Animal behaviour

## Abstract

Domestic animals are sensitive to human cues that facilitate inter-specific communication, including cues to emotional state. The eyes are important in signalling emotions, with the act of narrowing the eyes appearing to be associated with positive emotional communication in a range of species. This study examines the communicatory significance of a widely reported cat behaviour that involves eye narrowing, referred to as the slow blink sequence. Slow blink sequences typically involve a series of half-blinks followed by either a prolonged eye narrow or an eye closure. Our first experiment revealed that cat half-blinks and eye narrowing occurred more frequently in response to owners’ slow blink stimuli towards their cats (compared to no owner–cat interaction). In a second experiment, this time where an experimenter provided the slow blink stimulus, cats had a higher propensity to approach the experimenter after a slow blink interaction than when they had adopted a neutral expression. Collectively, our results suggest that slow blink sequences may function as a form of positive emotional communication between cats and humans.

## Introduction

For companion animals, humans act as key social partners, with these species often spending more time with humans than conspecifics^1^. The ability to engage in interspecific communication, not only by reading human-given cues but also by producing signals directed at humans, would have obvious adaptive advantages for domestic species, allowing the exchange of important social information. A number of domestic animals have been shown to use human-given cues to derive contextual information and perform tasks. Dogs are highly skilled in reading human gestural cues to locate hidden food^2,3^, and can differentiate between the attentive states of handlers when making choices^4–6^. More recent studies have revealed that dogs can also gain information through the use of human cues to emotion^7–10^. Horses, pigs and goats also all perform successfully in following certain human-given cues in object choice tasks^11–13^, and horses and goats can discriminate between different human emotions^14–16^, adjusting their behaviour in functionally relevant ways (in horses^17^). Interspecific communication is not one sided either—some domestic animals can also direct communicative behaviour towards humans. Human-directed gazing behaviour has been suggested to be a form of referential and intentional communication^18,19^ and is seen in dogs as young as 2 months old^20^, as well as in other domestic species, such as horses^21^ and goats^22^.

It is notable that the socio-communicative abilities of another key species in the human household, the domestic cat (*Felis Catus*), have been relatively understudied—perhaps because of the cat’s solitary ancestry^23^. However, research assessing human-directed gazing by cats in a social referencing paradigm found that most cats do look to their owners when faced with an ambiguous situation^24^. In addition, domestic cats have been shown to perform equally as well as dogs in object choice tasks using human pointing^25^, and only slightly worse than dogs in tasks that require human-directed attention-getting to obtain food^23^. Cats have also been shown to attract and manipulate human attention effectively in the auditory modality, through solicitation purring^26^, as well as discriminate their name from other words, even when unfamiliar humans are calling^27^. There is evidence that they also display specific facial actions during negative emotional contexts with humans compared to the same emotional contexts without human interaction^28,29^. Furthermore, there is circumstantial evidence that cats may be sensitive to human emotional cues, displaying more allo-rubbing (a communicative signal of affiliation in cats^30^) toward owners in a depressed mood^31^. Cats also alter their behaviour in different ways, depending on the valence of the emotional cues presented by a familiar human^32^. These findings collectively suggest that the cat may be an interesting model for further investigation of inter-specific communication, particularly in the context of emotional communication with humans. This line of study has particular relevance for enhancing the cat–human bond and feline welfare.

**Figure 1:**
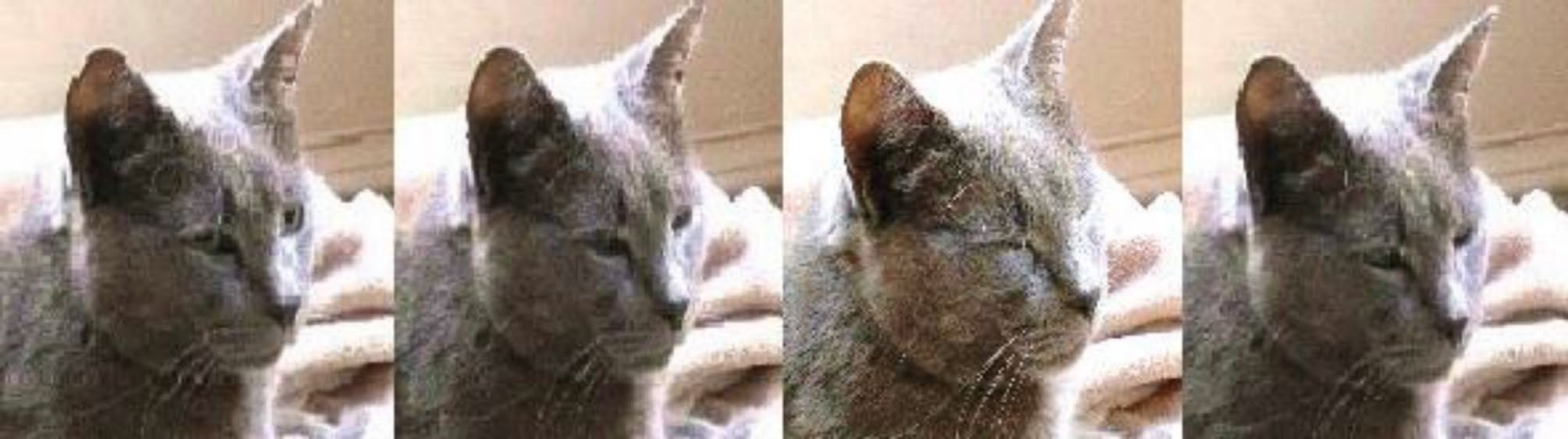
The cat slow blink sequence, starting from a neutral face moving to a half blink, then towards eye closure and then eye narrowing expression.

One common anecdotally acknowledged yet subtle behavioural display that cats appear to direct at humans is the slow blink sequence (see also^33^). Slow blink sequences involve a series of half-blinks (where the eyelids move towards each other without ever fully closing the eye^34^) followed by either prolonged narrowing of the eye aperture or a full eye closure (see Fig. 1). Anecdotal evidence and personal observations suggest that the slow blink sequence can be used as a method of cat–owner communication, and is said to occur in calm, positive contexts^35,36^. Interestingly, narrowing of the eyes, the main characteristic of the slow blink sequence, also features in the positive emotional displays of some other species, including the play and consummatory faces of canids^37^, in horses and cows during stroking^38–40^ and the human Duchenne smile^41^, and might therefore be a positive emotional indicator in cats.

We performed two experiments with the aim of exploring the communicatory significance of slow blink behaviour in human-cat interactions. We first examined whether cats respond to human-initiated slow blink stimuli with slow blink sequences of their own (Experiment 1). We then investigated whether cats were more likely to approach an unknown experimenter after a slow blink interaction (Experiment 2). Across the experiments, the slow blink sequence was compared with two controls: no human interaction, and a neutral face.

## Materials and methods

### Experiment 1

#### Subjects

A total of 21 cats from 14 different households were included. Fourteen different owners participated in this experiment. Ten of the cats were male and 11 of the cats were female, cat age ranging from an estimated 0.45–16 years (M = 7.05, S.D. = 4.59). All cats were housed indoors with outdoor access and had been living with their owners for a minimum of 3 months. Due to the nature of the study, partially blind/visually impaired cats or cats with medical issues involving the eyes were not included. All subjects were filmed during the slow blink stimulus, and this was counter-balanced with the no human interaction condition. Three dyads were outliers and excluded from subsequent analyses (> 2 standard deviations from the mean), thus the final analyses included 18 cat–human dyads.

#### Procedure

Cats were individually tested in a familiar room within their home. The home environment is more comfortable for the cats than laboratory based contexts, increasing the ecologically validity of the testing conditions. The experimenters (RS and TH) demonstrated the slow blink action, an eye closure (lasting more than 0.5 s), and gave advice verbally on how to perform the facial actions associated with slow blinking. Additional exerts from the FACS manual were provided if owners required more detailed descriptions of related movements (see Supplementary Note). The experimenter also gave verbal instructions on the intensity of these actions and then asked the owner to produce the slow blink movements to check that the cue was appropriate, giving corrections if necessary. The cat was present at all times throughout the experiment. Once the cat had settled down in one place, the experimenter asked the owner to either sit approximately 1 m in front of their cat or not to interact with their cat for the duration of the trial, depending on the condition. A Sony DSC-HX9V video camera was positioned 1.0–1.5 m in front of the owner to record their facial expressions, and a second Canon G9 video camera was placed the same distance in front of the cat. The experimenter was situated behind the video camera that recorded the cat’s facial expressions.

Paired slow blink stimulus and no human interaction conditions were counterbalanced across cats, with each cat being presented with each condition once. Before delivering each slow blink stimulus, owners were asked to ensure the cats were attentive and if they were not, to gain their cat’s attention. Once the cat gave direct eye contact, owners performed the slow blink action. Owners were asked to repeat this procedure until the experimenter indicated the end of the trial. Trials varied slightly in length due to the cat’s motivation to participate in the interaction but were 2 min maximum or ended when the cat walked away. On average trials lasted 62.75 s (s.e.m = 8.71; range: 19.14–120) long and the average owner’s stimulus delivery was at a rate of 14.58 (s.e.m = 0.03; range: 3–30.6) slow blink stimulus eye movements per minute. During the no human interaction condition, the owner remained in the room with the cat, but did not sit in front of or interact with the cat—owners were free to speak to the experimenter at this time. Mean trial length for the no human interaction control condition was 59.86 s (s.e.m = 8.50; range: 21.03–120).

#### Behavioural coding

Cat’s eye movements were coded using actions defined in CatFACS, an anatomically based system designed to objectively measure facial actions based on their underlying muscle movements^34^. One additional code (not specifically accounted for in CatFACS), ‘eye narrowing’, was included in the current study to take into account situations where the eye aperture was held partially closed for at least 2 frames (0.08 s) rather than returning to the neutral eyelid position in a continuous movement, as in the half blink (see Table 1). The eye narrowing and eye closure movements of the owner were also coded. Eye movements of both cats and humans were coded by one researcher (TH), who had been present in only a proportion of the original trials and was blind to the conditions for the cats’ eye movements; human facial expressions could not be coded blind, as the condition was obvious from the owner’s facial expression. A second independent coder analysed a random 25% of the videos. The independent coder was familiar with the research aims and was completely blind to condition (and had not been present during any of the trials). Both researchers were certified CatFACS coders and the inter-observer reliability on coding the presence of all cats’ and owners’ eye movements yielded a Cronbach’s alpha of 0.9, which is deemed a good level of inter-observer reliability. Figure 1 shows the cat slow blink sequence taken from video frames of one of the subjects; video available as Supplementary Video.

**Table 1:** Cat and human eye movements and corresponding FACS action units.

Code name	Facial action unit	Description of code
Cat half blink	AU 147	One of the eyelids (upper or lower) moves towards the other without ever closing the eye. It can occur in only one eye. It may occur in a succession of movements or one movement only
Cat eye closure	AU 143	The upper and lower eyelids move towards each other and cover the eye completely. The eye has to remain closed for more than half a second. It can occur in only one eye
Cat eye narrowing		The upper and lower eyelids are held half closed. This is a prolonged version of AU147
Cat blink	AU 145	The upper and lower eyelids move towards each other and cover the eye completely. The eye has to open within half a second. It can occur in only one eye
Cat eye closures due to movement		When a cat closes its eyes due to rubbing against a surface, scratching, yawning or any other movement that would naturally cause the eyes to narrow or close
Human eye closure	AU 43	The upper and lower eyelids move towards each other and cover the eye completely. The eye has to remain closed for more than half a second
Human eye narrowing		The upper and lower eyelids are held half closed. The eye aperture is held partially closed for at least 2 frames, as in Cat Eye Narrowing

See^34^ and^42^ for descriptions and photographs of the actions described.

#### Statistical analysis

Due to trials varying in length, the rate of each cat’s individual eye movements (half-blink, blink, eye closure and eye narrowing) was calculated by dividing the total number of a particular eye movement by the total length of the trial in seconds. The resulting rates of the individual cat eye movements (as the response variable) were then compared across the slow blink and the no human interaction conditions using a series of linear mixed models conducted in R version 1.2.5001 using the lmerTest package and the number of iterations set to maximum. In addition to the fixed factor of Condition (control versus experimental), the Number of Cats in the Household, Cat Sex and Cat Age were included as fixed factors. To account for some owners having more than one cat as a subject, Cat Identity nested within Household was included as a random factor. For each response variable (blink, half-blink and narrowing), a null model and global model was run. Factors with little or no predictive value were systematically removed from the global model to produce the best-fit model based on the Aikaike information criterion (AIC). Model selection tables and details of the human eye movements across conditions can be found in the Supplementary Data. If individual eye movements occurred in less than 5 cats, statistical analyses could not be performed for this movement.

### Experiment 2

#### Pilot trials

Pilot trials were conducted which included a control condition, involving a neutral facial expression with direct eye contact toward the cat. These trials indicated that in cats, as in some other species (for a review see:^43^), individuals may perceive direct eye contact from humans as threatening. Thus, we modified the control condition in Experiment 2 to a neutral face without direct eye contact being made.

#### Subjects

In total 24 cats were recruited from local online advertisements. Twelve cats were male and 12 cats were female, cat age ranging from an estimated 1–17 years old (M = 6.00, S.D. = 4.78). All cats were housed indoors with outdoor access. As in Experiment 1, partially blind/visually impaired cats or cats with medical issues involving the eyes were not included in this study. Cats were naïve, they were not involved in experiment 1. The cats included in the final analyses were from 8 different households. All subjects were recorded under both slow blink stimulus and neutral face conditions, and the order of conditions was counterbalanced between subjects. Six cats were excluded from the subsequent analyses due to outliers in the data (> 2 standard deviations from the mean rate of cats’ eye movements), and therefore the final analyses included 18 cats.

#### Procedure

The experimenter (JF) avoided contact with the cat before trials began, interacting only with the owners. Before testing, owners were encouraged to uphold the normal atmosphere of the household, and were allowed to talk and move around as they pleased at this stage. During trials they were stationary and did not interfere with the cat but did sometimes talk. As in Experiment 1, cameras were set up once the subject had settled down, allowing cats to habituate to the presence of the camera equipment. Video footage was obtained using a wide-angle lens Panasonic HC-X920 camera placed 1.0–1.5 m away from where the cat had settled and an additional Sony DCR-SR37 camera 1.0–1.5 m in front of the experimenter. A 2-min baseline was captured to record the cat’s typical behaviour. The experimental trials began with the experimenter offering the cat a flat hand with palm faced upwards whilst sat or crouched directly opposite the cat. If the cat was not attentive, the experimenter called the cat’s name. This action was carried out to observe the cat’s baseline level of approach tendency. After a few seconds, the experimenter retracted her hand and either adopted a neutral expression without eye contact or began performing the slow blinking stimulus. To standardise the experimenter’s head position between the neutral stimulus and the slow blink stimulus, the experimenter simply looked away slightly to the side of the cat during the neutral face without eye contact condition.

Delivery of the slow blink stimulus was identical to that in experiment 1. Trials lasted a minute, after which the experimenter again offered her hand for a few seconds, as an approach invitation stimulus. The experimenter’s hand was offered for an average of 3.71 s, and there were no significant differences in the length of time the experimenter offered her hand in each condition (Z =  − 1.02, *p* = 0.31). Cats’ responses to the approach invitation were measured and the retraction of the experimenter’s hand signalled the end of the trial. In between trials, there was an interval of approximately 2 min in order to give cats a break from social interaction and to avoid any carry-over effects across trial types which may affect the approach response.

#### Behaviour coding

Behavioural coding was the same as for experiment 1 (see Table 1), with the exception that the normal reflexive blinking action was omitted from the coding scheme, as normal maintenance blinking had not contributed to the differences found in experiment 1 and did not appear to be part of the cat slow blink sequences. Codes for approach behaviour were also included which consisted of Approach, Neutral and Avoid. Approach was defined as any head or body movement towards the proffered hand, Avoid as any head or body movement away, and Neutral as no change in movement. Experiment 2 also incorporated a factor that accounted for eye responses that could have occurred as a result of the experimenter calling the cat’s name to gain their attention during the trials, these were controlled for by excluding any cat eye movements made within half a second of an experimenter’s call, in the absence of an experimenter eye closure.

#### Statistical analysis

All trials in experiment 2 lasted the same length of time (1 min), therefore number of the cat’s eye movements was used directly in the analyses, rather than being converted into rates as in experiment 1. As in experiment 1, the resulting scores for the individual cat eye movements (half-blink, eye closure and eye narrowing) were compared across Condition (control vs. experimental) using a series of linear mixed models with the addition of the fixed factors of Number of Cats in the Household, Cat Sex and Cat Age. To account for some owners having more than one cat as a subject, cat identity nested within household was included as a random factor. For each response variable (half-blink, eye closure and narrowing), the best-fit model was chosen based on the AIC. Model selection tables and details of the human eye movements across conditions can be found in the Supplementary Data. Wilcoxon signed-rank test was used to examine differences in tendency to approach across the slow blink stimulus and neutral condition (coded as 1 = avoid, 2 = neutral, and 3 = approach).

### Ethical statement

This research follows the Association for the Study of Animal Behaviour Guidelines for the Use of Animals (Animal Behaviour, 2006, 71, 245–253) and all experimental protocols were approved by the University of Sussex Ethical Review Committee (ERC), reference number: Non-ASPA—Nov2013. The experiments were carried out in accordance with the relevant guidelines and regulations. Informed consent for participation and publication of identifying information and images in an online open-access publication was gained from all relevant cat owners (Experiment 1 and 2) and no participants were under the age of 18.

## Results

### Experiment 1

**Figure 2:**
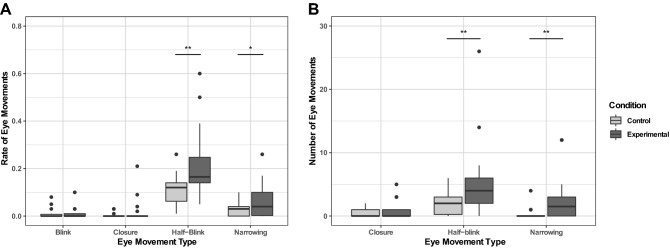
(**A**) Boxplot showing rate of cats’ eye movements during slow blink stimulus (experimental) condition and no human interaction (control) condition in Experiment 1. (**B**) Boxplot showing number of cats’ eye movements during the slow blink stimulus (experimental) condition and neutral face (control) condition in Experiment 2. * = *p* < 0.05, ** = *p* < 0.01.

For the eye narrowing movement, the best-fit model contained the fixed factor Condition (experimental versus control) only. During the slow blink stimulus (experimental) condition the rate of eye narrowing was significantly higher than in the no human interaction (control) condition (Experimental M = 0.06 ± 0.07, Control M = 0.03 ± 0.03, z =  − 2.64, *p* = 0.017; see Fig. 2A). For the response variable half-blink, the best-fit model contained the factors Condition, Sex and Sex * Condition. During the slow blink stimulus (experimental) condition the rate of eye narrowing was significantly higher than in the no human interaction (control) condition (Experimental M = 0.21 ± 0.15 (SD), Control M = 0.15 ± 0.06, z = 3.45, *p* = 0.003). There was also a significant interaction of Sex * Condition, with male cats showing a significant increase in the rate of half-blinks in the experimental condition compared to the control and the female cats not showing a strong effect of condition (Male Experimental M = 0.30 ± 0.16, Male Control M = 0.13 ± 0.07; Female Experimental M = 0.13 ± 0.06, Female Control M = 0.10 ± 0.05, z = 2.51, *p* = 0.02). There was no effect of Cat Age or Number of Cats in the Household for the half-blink or eye narrowing movements. For the standard blinks, there were no significant predictors and the best-fit model was the null model, suggesting that normal (reflexive) blinks were not affected by the experimental condition. Only 4 cats showed complete eye closures in the slow blink condition and 3 in the no human interaction condition—so analyses could not be performed on this variable (see Fig. 2A).

### Experiment 2

**Figure 3:**
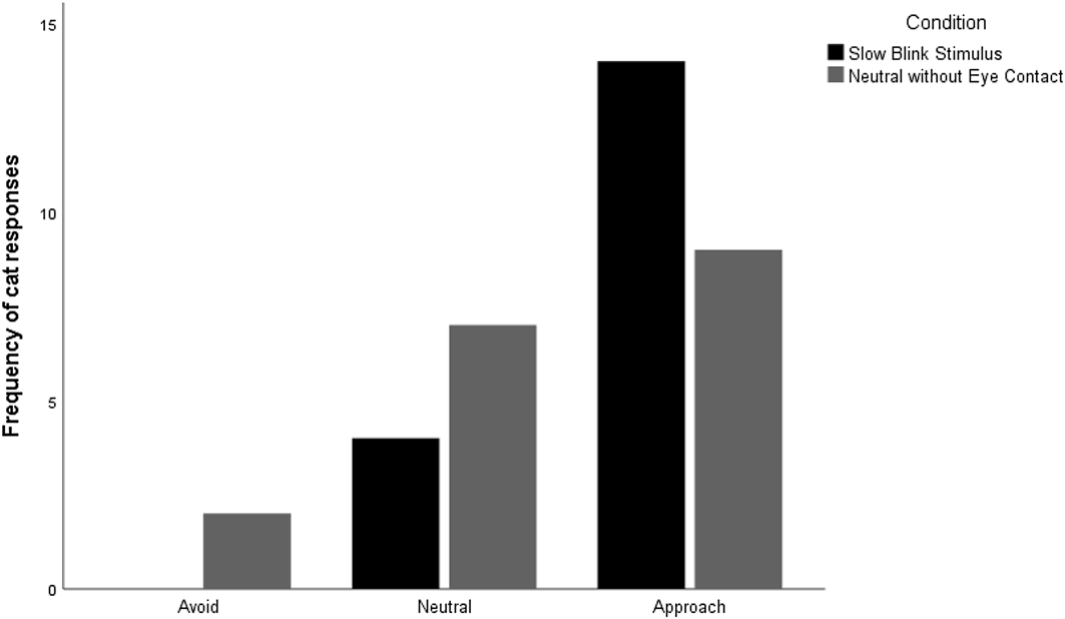
Frequency of Cats’ Responses (Avoid, Neutral, or Approach) in relation to condition (Slow Blink Stimulus or Neutral). Cats had a significantly higher approach score following the slow blink condition compared to the neutral condition (*p* = 0.035).

For the half-blinking and eye narrowing movements, the best-fit models contained only the fixed factor Condition (experimental versus control). During the slow blink stimulus (experimental) condition the rate of half-blinks and eye narrowing were significantly higher than in the neutral face (control) condition (*Half blinks*: Experimental M = 5.33 ± 6.10, Control M = 1.94 ± 1.77, z = 2.92, *p* = 0.007; *Eye narrowing*: Experimental M = 2.78 ± 3.73, Control M = 0.33 ± 0.97, z = 3.35, *p* = 0.002; see Fig. 2B). There was no effect of Sex, Age or Number of Cats in the Household for either movement. For the eye closure movement, the best-fit model contained the factor Cat Age (z =  − 2.35, *p* = 0.02), with older cats performing more eye closures. Cats also had higher approach scores to the hand of an experimenter after the slow blink stimulus presentation (M = 2.78 ± 0.43) than after the neutral face (M = 2.39 ± 0.70), z = 2.11, *p* = 0.035 (see Fig. 3).

## Discussion

This study is the first to experimentally investigate the role of slow blinking in cat–human communication. Our results not only describe the specific movements involved in cat slow blink sequences but also produce several strands of evidence which collectively suggest that cats respond to a human giving a slow blink stimulus by producing eye narrowing movements of their own. Firstly, cats deliver more eye narrowing movements when their owners slow blink at them than when the owner is present in the room but not delivering this stimulus. Secondly, when an unfamiliar experimenter gives the slow blink stimulus compared to adopting a neutral face, cats respond with a higher frequency of eye narrowing movements themselves. In addition, the study produces evidence that cats perceive human slow blinking in a positive way, as subjects prefer to approach an experimenter after a slow blink interaction has occurred, compared to when the experimenter adopts a neutral facial expression without direct eye contact with the cat. This is in accordance with previous anecdotal reports of this behaviour as signalling relaxation in cats^33,35^.

Approach-avoidance has long been used to measure the primary motivation systems that are key to animals’ emotional responses^44^, where an individual’s approach is taken to indicate that stimuli are perceived as pleasant^45^. The propensity of cats to approach humans following a slow blink stimulus could be because the slow blink sequence behaviour itself elicited an inherently positive emotional state in the cats or because they simply perceived the unfamiliar experimenter as pleasant after a slow blink stimulus, having learnt this from prior exposure. It is notable that, anecdotally, cats are often seen to initiate slow blink interactions themselves^33^, suggesting that the signalling interaction itself is something they are motivated to engage in. It is also relevant to note that the slow blink stimulus shares certain features with the Duchenne smile (the genuine smile in humans^41^), as well as responses in other mammals to positive emotional contexts (during grooming, in horses:^39^; in cows:^38^; in sheep:^46^; during feeding in cows:^40^; and during relaxation (anecdotally) and play in canids:^37,47^)—specifically the narrowing of the eyes is an integral part of all of these signals. Thus our results could suggest that cats share some of the same features of positive signalling which have been found in a wide range of animals, including humans. However, since the experimenters did not make direct eye contact with the cats in Experiment 2 during the neutral condition, it remains possible that the experimenter’s gaze direction or attentional state may potentially have affected cats approach responses and this would be a factor to investigate in future work. It would be useful in future studies to explore the potential presence and function of this behaviour in conspecific communication as well as in cat–human signalling—and also consider its occurrence in enculturated or socialised, captive felids.

Our study used both owners and an unknown experimenter to deliver the slow blink stimulus. In doing this we found that both owners and an unfamiliar other could elicit a slow blink sequence in the cats. These findings somewhat contrast with Galvan and Vonk’s^32^ study which found sensitivity of cats to cues of emotion from owners but not an unknown experimenter. Differences in methodology possibly led to these differing results. In particular, the use of CatFACS^34^ for analysing cats’ facial behaviours in our study may have allowed more detailed responses to be observed. One limitation of Experiment 1 was the presence of individual variation between owners in performing the slow blink stimulus, despite instructions having been provided. Variation was also present in the slow blink displays given by the owners through inter-owner variability in a range of cues, including the extent to which they talked to the cat and in facial features such as whether they wore glasses. Also, as the cat was in the room in the control trials without the owner specifically interacting with it, there was variation in the distance between owner and cat here that we were unable to quantify. This added noise to the data, making it potentially difficult to determine which signals the cats were responding to. In Experiment 2, we sought to overcome this by having an experimenter perform the facial stimuli across cats in order to standardize the presentations. The use of an unfamiliar experimenter in Experiment 2 also allowed us to investigate cats’ appraisal of a person performing slow blinking. Our experiments were naturalistic, conducted in the cat’s home environment, so it was not possible to control exactly where the cat was resting or the precise distance from the cat to the owner’s eyes without disrupting the subject. Variation in this is true to real-life interactions between owners and their cats and would be expected to add noise to the experiment—but also robustness—and would not systematically bias the results. However, it would be interesting and worthwhile to replicate the experiments in a lab setting.

It could be argued that cats have developed slow blink behaviours because humans appear to perceive slow blinking as positive and cats may have previously been reinforced by their owners for responding to slow blink sequences. Including an unfamiliar human therefore reduced such reinforcement effects, although the cats may still generalize across humans. Further research on slow blinking behaviour in cats could consider whether this behaviour is an evolved trait or learnt over time. It is also possible that slow-blinking in cats originated as a mechanism to interrupt an unbroken stare, which is potentially threatening in social interactions^43^; this could then have been elaborated by a combination of selection and learning in the domestic environment. Understanding specific ways in which cats and humans may interact positively, such as through eye narrowing movements, can enhance public understanding of cats and feline welfare, particularly considering the close bond cats and humans share^48,49^. From the current study, the slow blink sequence appears to be an indicator of positive emotion in cats. Identifying observable indicators of positive emotions has practical benefits for the welfare of animals by providing assessment markers of an individual’s current welfare and pointing to behaviours that can be promoted to produce a better quality of life^50^. However, further research would be necessary before coming to firm conclusions about the emotional state associated with slow blinking/responding to slow blinking.

In summary, our study provides the first systematic investigation of the role of slow blink behaviour in cat–human communication. We show that slow blink interactions appear to be a positive experience for cats, and may be an indicator of positive emotions. Such findings could potentially be used to assess the welfare of cats in a variety of settings, including veterinary practices and shelter environments as well as enhancing cat–human communication in the human home. Socio-cognitive abilities of cats are an under-studied area, and future research on cat behaviours, such as slow blinking, could enhance our understanding of interspecific communication and the ways in which domestication has shaped the social behaviour of an ancestrally solitary species.

## Supplementary information

Supplementary Information 1.

Supplementary Information 2.

Supplementary Information 3.

Supplementary Information 4.

Supplementary Information 5.
