# Supplementary material for: The role of cat eye narrowing movements in cat–human communication
Source: Sci Rep. 2020 Oct 5;10:16503. doi: 10.1038/s41598-020-73426-0 (PMC7536207; doi:10.1038/s41598-020-73426-0)
Supplement: Supplementary file 1 — Supplementary Information 1. [file 41598_2020_73426_MOESM1_ESM.pdf]

The role of cat eye narrowing movements in cat-human communication

Tasmin Humphrey[1]*, Leanne Proops[1,2], Jemma Forman[1], Rebecca Spooner[1], Karen McComb[1]*

Affiliations

1. Mammal Vocal Communication and Cognition Research Group, School of Psychology, University of Sussex, Brighton, BN1 9QH, UK
2. Centre for Comparative and Evolutionary Psychology, Department of Psychology, University of Portsmouth, Portsmouth, PO1 2DY, UK

* Correspondence to T.Humphrey@sussex.ac.uk & karenm@sussex.ac.uk

Supplementary Note:

Experiment 1.

Instructions for owners:

Verbal instructions

With a positive yet calm expression, close your eyelids slowly and purposefully whilst drawing your cheeks upward. Try to slow this action down to around 2-3 seconds.

Be careful not to also lower your eyebrow or wrinkle your nose.

Additional FACS instructions made available

AU 12 Pull the corners of your mouth up and backwards into a U shape (deepening the crease to the outside of the mouth) this will raise the adjacent skin upward.

a. The cheeks are pushed upward which should also narrow the eye aperture

b. The deepening of the crease below your eyes is more evident

c. The skin below the lower eyelid bags

d. Produces crow's feet at eye corners

AU 6 Ensure that the skin from the temple and cheeks is drawn toward the eye – as the outer band of muscle around the eye constricts (similar effects to that of AU12)

AU 7 Tense your eyelids but not enough to close your eyelids completely. Do it as weakly as you can.

[If you have difficulty, think about narrowing your eye aperture to a slit so that you can see your eyelashes. Be careful you are not also lowering your eyebrow (AU 4). Be careful you are not also wrinkling your nose (AU 9).]

More complex definition for those who find it difficult (to be spoken through by the experimenter)

AU 6: Concentrate on lifting your cheeks without actively raising up the lip corners (that is AU 12).

The muscle underlying AU 6 (like that responsible for AU 7) circles the eye orbit, but it has a larger circumference that extends into the eyebrow and below the lower eye furrow. Action Unit 6 pulls skin towards the eye.

1. Draws skin towards the eye from the temple and cheeks as the outer band of muscle around the eye constricts.
2. Raises the infraorbital triangle, lifting the cheek upwards.
3. Pushes the skin surrounding the eye towards the eye socket, which can narrow the eye aperture, bag or wrinkle the skin below the eye, and push the eye cover fold down and/or change its shape.
4. May cause crow's feet lines or wrinkles to appear, extending radially from the outer corners of the eye aperture.
5. Deepens the lower eyelid furrow. (Apparent in image w6 more than in 6.)
6. May lower lateral portion of the eyebrows to a small extent (in image w6 not 6).
7. A strong AU 6 may:
a. Make evident or deepen the nasolabial furrow.
b. Raise the outer portions of the upper lip to a small extent.
c. Make evident or deepen the infraorbital furrow, so that this wrinkle runs across the top of the infraorbital triangle in a straight or crescent-like shape.

AU 7: This movement is fairly easy to do. Tense your eyelids but not enough to close your eyelids completely. Do it as weakly as you can. If you have difficulty, think about narrowing your eye aperture to a slit so that you can see your eyelashes. Be careful you are not also lowering your eyebrow (AU 4). Be careful you are not also wrinkling your nose (AU 9). Be careful you are not also raising your cheeks (AU 6).

AU 12: This movement is an easy to do. Smile. Imitate image 12i. Holding a weak version on your face, slowly increase the extent of action and watch as your face begins to resemble 6+12ii and 6+12+25ii.

1. Pulls the corners of the lips back and upward (obliquely) creating a U shape to the mouth
2. Deepens the nasolabial furrow, pulling it laterally and up. The skin adjacent to the nasolabial furrow is raised up and laterally.
3. In a weak to moderate 12, there is some raising of the infraorbital triangle and there may be some deepening of the infraorbital furrow.
4. In a strong action, one or more of the following:
   a. The infraorbital triangle push upwards is more evident.
   b. The infraorbital furrow deepening is more evident.
   c. Bags the skin below the lower eyelid.
   d. Narrows the eye aperture by pushing up the cheek and skin below the lower lid.
   e. Produces crow's feet at eye corners.
   f. May raise and widen the nostrils.

g. May flatten and stretch the skin on the chin boss

Experiment 2.

Facial Expression Instructions for Experimenter

Slow Blink Stimulus

For the slow blink stimulus, purposefully and slowly lower your eyelids whilst drawing your cheeks upward, toward the eye.

Try to perform this expression slower than you usually would in a conversation (approximately 2-3 seconds). Be careful not to also lower your eyebrow or wrinkle your nose.

Neutral Face Control

For this action try to release from muscle tension in the face. To do this it may help to relax and calm your thoughts, as if in a meditative state. Gaze slightly to the side of the cat whilst keeping your face forward towards the cat.

Be careful not to hold the expression too strongly, to avoid looking stern whilst performing this facial expression.
