# Supplementary material for: The role of cat eye narrowing movements in cat–human communication
Source: Sci Rep. 2020 Oct 5;10:16503. doi: 10.1038/s41598-020-73426-0 (PMC7536207; doi:10.1038/s41598-020-73426-0)
Supplement: Supplementary file 5 — Supplementary Information 5. [file 41598_2020_73426_MOESM5_ESM.docx]

**The role of cat eye narrowing movements in**

**cat-human communication**

Tasmin Humphrey^1^*, Leanne Proops^1,2^, Jemma Forman^1^, Rebecca Spooner^1^, Karen McComb^1^*

### Affiliations

#### Mammal Vocal Communication and Cognition Research Group, School of Psychology, University of Sussex, Brighton, BN1 9QH, UK

#### Centre for Comparative and Evolutionary Psychology, Department of Psychology, University of Portsmouth, Portsmouth, PO1 2DY, UK

* Correspondence to [T.Humphrey@sussex.ac.uk](mailto:T.Humphrey@sussex.ac.uk) & [karenm@sussex.ac.uk](mailto:karenm@sussex.ac.uk)

**Model Comparisons**

- **Experiment 1 Rate of eye movements: blinks**

| **Model terms** | **AIC** |
| --- | --- |
| **NULL:** 1 + (1\|household_number/cat_number) | **-163.4** |
| **GLOBAL:** cats_household + sex + age + condition + (1\|household_number/cat_number) | -157.1 |
| cats_household + sex + age + (1\|household_number/cat_number) | -159.1 |
| cats_household + sex + (1\|household_number/cat_number) | -160.9 |
| sex + (1\|household_number/cat_number) | -162.3 |

- **Experiment 1 Rate of eye movements: half blink**

| **Model terms** | **AIC** |
| --- | --- |
| **NULL:** 1 + (1\|household_number/cat_number) | -41.5 |
| **GLOBAL:** cats_household + sex + age + condition + (1\|household_number/cat_number) | -48.5 |
| sex + age + condition + (1\|household_number/cat_number) | -50.4 |
| sex + condition + (1\| household_number/cat_number) | -52.3 |
| sex + condition + sex*condition + (1\|household_number/cat_number) | **-55.8** |
| sex + (1\| household_number/cat_number) | **-**46.3 |
| condition + (1\| household_number/cat_number) | -46.5 |

- **Experiment 1 Rate of eye movements: narrowing**

| **Model terms** | **AIC** |
| --- | --- |
| **NULL:** 1 + (1\|household_number/cat_number) | -97.1 |
| **GLOBAL:** cats_household + sex + age + condition + (1\|household_number/cat_number) | -95.9 |
| cats_household + age + condition + (1\|household_number/cat_number) | -97.9 |
| cats_household + condition + (1\|household_number/cat_number) | -99.7 |
| condition + (1\|household_number/cat_number) | **-101.0** |

- **Experiment 2 Rate of eye movements: closure**

| **Model Terms** | **AIC** |
| --- | --- |
| **NULL:** 1 + (1\|household_number/cat_number) | 102.6 |
| **GLOBAL:** cats_household + cat_age + sex + condition + (1\|household_number/cat_number) | 106.2 |
| cat_age + sex + condition + (1\|household_number/cat_number) | 104.2 |
| cat_age + condition + (1\|household_number/cat_number) | 102.4 |
| cat_age + (1\|household_number/cat_number) | **101.0** |

- **Experiment 2 Rate of eye movements: half Blink**

| **Model Terms** | **AIC** |
| --- | --- |
| **NULL:** 1 + (1\|household_number/cat_number) | 217.5 |
| **GLOBAL:** cats_household + cat_age + sex +  condition + (1\|household_number/cat_number) | 216.2 |
| cat_age + sex + condition + (1\|household_number/cat_number) | 214.3 |
| cat_age + condition + (1\|household_number/cat_number) | 212.8 |
| condition + (1\|household_number/cat_number) | **212.1** |

- **Experiment 2 Rate of eye movements: narrowing**

| **Model Terms** | **AIC** |
| --- | --- |
| **NULL:** 1 + (1\|household_number/cat_number) | 185.1 |
| **GLOBAL:** cats_household + cat_age + sex + condition + (1\|household_number/cat_number) | 183.3 |
| cats_household + cat_age + condition + (1\|household_number/cat_number) | 181.4 |
| cats_household + condition + (1\|household_number/cat_number) | 179.4 |
| condition + (1\|household_number/cat_number) | **177.6** |
